# Research and application of tourism management in the IoT industry under the background of sustainable blockchain

**DOI:** 10.1016/j.heliyon.2024.e35893

**Published:** 2024-08-09

**Authors:** Zhenhua He, Wenwen Yu, Lifeng Chen

**Affiliations:** a School of Logistics and Transportation and Tourism, Jiangsu Vocational College of Finance and Economics, Huaian, 223003, Jiangsu, China; b School of Global Business Administration, Anyang University, Anyang, 14028, Gyeonggi, South Korea; c School of Business, Hangzhou City University, Hangzhou, 310015, Zhejiang, China; d School of Public Affairs, Zhejiang University, Hangzhou, 310058, Zhejiang, China

**Keywords:** Tourism management, Sustainable blockchain, Distributed technology, Internet of things

## Abstract

In the contemporary era, there is a heightened awareness of the significance of a spiritual life, and travel is a popular choice for many. The Internet is driving rapid diversification in people's travel choices. At the same time, the traditional tourism industry's Tourism Management (TM) model, which has been in place for decades, is no longer able to satisfy people's diversified choices for tourism. This is due to a number of factors, including the trust crisis caused by asymmetric information, the outdated nature of the TM model, and the insecure personal information that is shared between intermediaries. This paper discusses the application of sustainable blockchain technology in tourism management and sets up a tourism management system based on blockchain and the Internet of Things. It takes 20 scenic spots as examples to study the application of the TM model. This paper evaluates the TM model from four key aspects: tourist satisfaction, tourism infrastructure completeness, tourism consumption level and tourism service content richness. It compares the results with those of the traditional TM system. The experimental results are clear: tourist satisfaction at scenic spots 5, 10 and 15 in the sustainable blockchain TM mode is 68 %, 87 % and 71 % respectively, which is higher than that in the traditional TM mode. In the context of sustainable blockchain, the TM mode is the optimal approach for serving tourists and enhancing their travel experience. This study also assists Chinese tourism business operators in recognizing the potential impact of blockchain technology on the development of the tourism industry. They can then formulate strategies at an early stage to cope with the information technology changes in the era of the digital economy.

## 1 Introduction

Tourism is a strategic pillar industry of China's national economy, playing a clear role in driving economic development. Despite mounting economic challenges and a lack of internal enterprise motivation, tourism has demonstrated resilience and continued to grow. Tourism has been a significant contributor to economic growth, job creation and improvements in living standards and happiness. The advent of information and communication technology has led to a transformation of the tourism business model and management concept. This has resulted in a transformation of the interaction mode between enterprises and their stakeholders [1], thereby enhancing the quality of the tourism experience [2]. Meanwhile, digital transformation activities provide unparalleled intellectual support for the entire services process, including the retrieval of destination information, route planning, product booking, payment services and experience sharing [3]. In this context, the tourism industry can be distinguished by a high level of information intensity, and its innovative development is contingent upon the integration of the latest digital transformation.

The tourism industry has been transformed in recent years by the continuous development of new technologies such as big data, artificial intelligence, and blockchain. With the development of Internet VR technologies, people can enjoy a variety of tourism resources around the world without leaving your home [4]. The growth of the Internet gave birth to many third-party OTA platforms, but there are also many problems in the tourism industry. Today, travellers are mostly using traditional third-party OTA platforms for booking rooms, only to be told when they arrive that there is no room, or that there is a temporary price increase. The news of "passengers being kicked off the flight" is even more chilling. It is clear that the majority of people now buy local tourism products and specialities in tourism places. Unfortunately, many places take this opportunity to make inferior products and provide poor after-sales service, which results in consumers buying inferior products and specialities and suffering as a result. However, the smart contracts of blockchain technology offer innovative ideas for solving these dilemmas [5]. The tourism industry will benefit from the transparent database that blockchain provides. It is a tamper-proof system that cannot be manipulated. Once a transaction occurs, the blockchain records data in the reservation and payment system and can flexibly adjust pricing based on real-time tracking of supply and demand. Furthermore, consumers and service providers are exempt from the commission charged by the platform thanks to peer-to-peer transactions on the blockchain, saving them intermediate transaction fees and processes [6]. Furthermore, smart contracts can be more effectively linked to market forecasts and other services such as travel insurance on the blockchain, enabling better interaction with users on the blockchain [7]. This not only reduces unnecessary spending by consumers, but also enables travel companies to maximize revenue. The tourism industry is facing a significant challenge from the emerging technology of blockchain. The future is clear: the combination of blockchain technology and tourism will make the decentralisation of the tourism industry an inevitable trend in the development of tourism.

In gerenal, the tourism industry represents a significant component of the service sector, which has recognised the potential value of blockchain technology and the Internet of Things (IoT) in the context of digital transformation. And the convergence of blockchain and IoT technologies is becoming increasingly prevalent across a range of industries, driven by recent advances in both fields [8]. So the integration of sustainable blockchain technology and the tourism industry will undoubtedly enhance transparency and security, optimise the allocation and utilisation efficiency of tourism resources, and facilitate the sustainable development of tourism.

## 2 Literature review

As the tourism industry has developed, the theory and practice related to it have become increasingly sophisticated. Among these developments, the management and development of the tourism industry has emerged as a significant area of research. Valeri M and other scholars [9] have proposed the use of quantitative analysis methods to enhance the competitiveness of tourism destinations, employing Social Network Analysis (SNA). This method is essential for illustrating the data collection methods and techniques employed by other economic sectors. He presented a view of the relationship network, which may provide a powerful lever for tourism organisation managers to improve information flow and target the opportunities that these information flows may have the greatest impact on regulatory or commercial activities. Ghorbani A and other scholars [10] conducted a comprehensive study of organisations in the postmodern era with the objective of identifying a new type of organisation for the STO (Smart Tourism Organisation) theme. This novel organisational structure acknowledges the multifaceted dimensions of intelligence within the external environment and endeavours to cultivate these capabilities. In their analysis, Litvin S. W. and other scholars synthesised the findings of earlier published papers and evaluated the impact of electronic word-of-mouth (eWOM) on the hotel and tourism industries. He discussed the potential future developments, described the evolution of electronic word-of-mouth into an influential system, and particularly emphasised the role of mobile media as a platform for electronic word-of-mouth communication. He ultimately concluded that EWOM has fulfilled its promise to have a significant impact on the hotel and tourism industry and will continue to play an important role in hotel marketing for the foreseeable future [11]. In their research, Armenski T and other scholars investigated the fundamental aspects of destination competitiveness, examining the role of government and industry stakeholders in enhancing Serbia's competitive standing in the global tourism market. He employed both exploratory and confirmatory factor analysis. Finally, he identified the dimensions and proposed areas where public-private connections should be strengthened in order to enhance the competitiveness of Serbia's tourism industry [12]. These studies have significantly advanced the development of the tourism industry, offering insights into its management and development.

In recent years, there has been a great deal of research conducted on the application of blockchain technology, with the scope of this research gradually expanding to include the tourism industry. Wei D posited that digital technology is an indispensable tool for addressing the pressing challenges confronting the traditional tourism industry, particularly in enhancing the quality of service experiences and ensuring robust data storage security [13]. Melkic S and other scholars [14] sought to provide an answer by conducting a situational analysis of the impact of blockchain technology on the activities of all participants in tourism intermediaries. This analysis revealed that all parties would face various challenges in implementation and must carefully consider these challenges. The utilisation and pervasive comprehension of blockchain technology are still in their nascent stages. It is, therefore, imperative for those engaged in tourism research to assume an active role in investigating this field of enquiry. Given the dynamic nature of trends and their rapid development, analysis can facilitate a more nuanced understanding of this phenomenon, thereby enhancing the public's comprehension of research. Mucchi L and other scholars [15] employed museums as a case study to examine the utilisation of blockchain technology in cultural heritage organisations, and delineated the advantages and constraints of such technologies in cultural heritage loans. A qualitative approach, utilising case studies, was employed to introduce two development cases of blockchain-based tracking systems and blockchain smart contracts, along with their applications in museum cultural relic loan management. He concluded that the application of blockchain technology has facilitated the circulation of cultural goods, which is beneficial for the overall cultural supply, the attractiveness of museums, and possible tourism traffic. Nam K and other scholars have identified the key features of blockchain technology by combining smart cities and tourism frameworks, and have proposed suggestions on how blockchain technology would develop and impact the tourism industry [16]. The application of blockchain technology in the tourism sector has resolved the significant challenges confronting the traditional tourism industry and facilitated the advancement of the tourism industry.

It is anticipated that the market size of the blockchain industry will continue to expand, driven by factors such as national policy promotion, the advancement of foundational technology, and an increased demand for applications in downstream fields. It is imperative for the tourism industry to remain abreast of technological advancements and to respond expeditiously to emerging technologies, particularly in light of the central role that blockchain technology is poised to play in reshaping the traditional tourism industry. It is therefore imperative to investigate the potential applications of blockchain technology in the context of the tourism industry.

## 3 Current Situation of tourism and TM

### 3.1 Concept and nature of tourism

**Fig. 1:**
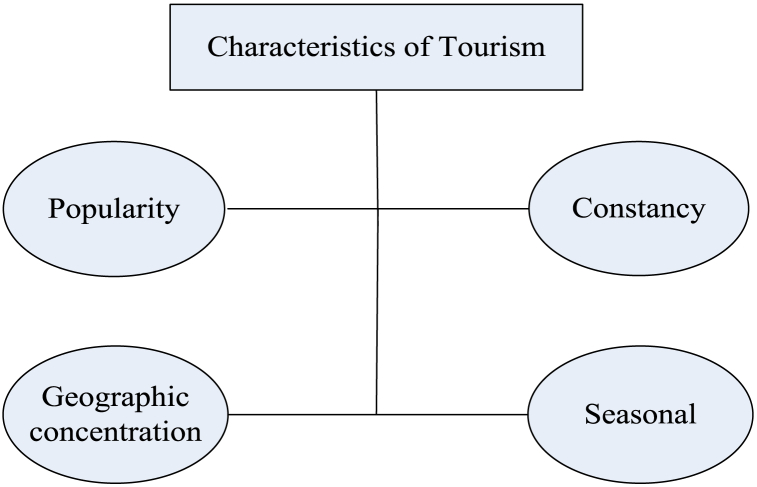
Characteristics of tourism activities.

The term 'tourism' is used to describe the traveling or staying behaviour of people who leave their usual place of residence for reasons other than moving or working. It also encompasses the various phenomena and relationships that arise from this behaviour [17]. The nature of tourism encompasses the following elements: Tourism is a social and cultural activity, characterised by people moving between different regions and involving economic, political and other issues. It can be defined as a leisure activity, a way for people to temporarily escape their daily lives and adopt a special way of life that is either short-term or long-term. The main aim of tourism is to obtain psychological pleasure through aesthetic and self-entertainment processes. It is the most fundamental activity of human society once a certain level of development has been reached. The characteristics of tourism activities are illustrated in Fig. 1.

### 3.2 Content of TM

The main content of TM [18] includes tourist activity management, tourism enterprise management, tourism market management, and tourism macro management.

#### *3.2.1* Tourist activity management

It is able to comprehend tourists' tourism needs, motivations, and the emergence and development of tourism behaviour, grasp the patterns of tourists' activities, and provide scientific guidance and management of tourists' behaviour. It must also be able to comprehend the content and characteristics of tourism services, to strengthen the management of service quality, service integrity and tourism safety throughout the tourism process, and to ensure the smooth and efficient development of tourism activities.

#### *3.2.2* Tourism enterprise management

The main content of this subject is the development management, strategy management, operation management and product development management of tourism enterprises. The purpose of this subject is to promote the development of the tourism industry.

#### *3.2.3* Tourism market management

This area of study aims to operate and manage the market environment and market development in which tourism enterprises are located. It is therefore necessary to have a certain understanding of the market system, competitive structure and market operation of the tourism industry. Furthermore, it is necessary to understand the ways and methods of market development and tourism promotion. In terms of macro-regulation of the tourism industry, it is necessary to make full use of market mechanisms, regulate and supervise the market, and make full use of the functions of industry organisations.

#### *3.2.4* Macro-level tourism management

TM institutions are a type of management organisation created with the specific objective of promoting the development of the tourism industry. It is essential to formulate legislation, policies and development plans related to tourism in order to guide the activities of tourists and the operation of tourism enterprises, improve the tourism market and promote the development of tourism. It is essential that people employ a range of legal, administrative, economic and other methods and means to regulate and manage the operation of the tourism industry in a manner that aligns tourism activities with the goals of economic and social development.

### 3.3 Functions of TM

The main functions of TM include planning, organization [19], leadership, and control. Its functions and significance are shown in Table 1.

**Table 1:** The function and significance of TM.

Function	Meaning
Plan	Develop a tourism development plan to guide tourists and tourism enterprises, improve the tourism market, and promote tourism development
Organization	Organize various management measures during the tourism process to ensure smooth activities for tourists
Lead	Unified development and management of tourism enterprises, leading the management of tourism enterprises
Control	Regulating and managing the operation of the tourism economy

### 3.4 Problems and solutions in TM

The online tourism platform is growing rapidly as the tourism industry develops at an incredible pace. The comprehensive online travel platform will provide consumers with one-stop, full-process services, including hotel ticket booking, travel guides, customised tours, travel products, and peripheral services. It attracts and gathers a large number of consumers and related upstream and downstream supplies. However, the travel market is also monopolised by major online travel platforms [20]. The platform has the pricing power of the channel, charges high commissions and service fees, and even makes differentiated pricing for consumers through big data means. This infringes the interests of relevant participants in the tourism industry and inevitably increases the cost of tourism consumption for consumers [21]. Monopoly is just one of the problems caused by centralisation in the process of informatisation and digital transformation of the tourism industry. Centralisation brings trust problems, tourism service evaluation fraud, malicious destruction of tourism public facilities, difficult data sharing of most systems, user privacy information disclosure, and difficult control of expensive goods.

Blockchain and tourism are a match made in heaven. They can effectively improve the efficiency of trusted data exchange and form a high-quality collaborative development mechanism. Upstream and downstream participants, such as tourism service agencies, government regulatory authorities, banks, communication operators, tourism transportation departments, and tourism area service agencies, respectively have different dimensions and sizes of tourism data. They are mutually dependent on each other in business, but they cannot communicate with each other and cannot realise data sharing. The tourism blockchain provides a new model for the opening up of tourism data. It allows business data to be jointly maintained by the alliance participants. This is done through privacy protection and encryption technology to make the data available on the chain invisible. It also uses smart contracts to automate the complex business logic. The tourism blockchain allows consumers, service providers, regulatory authorities and others to complete business directly, solving the trust problem, eliminating the high grey cost in the middle and effectively lowering the cost.

Blockchain and tourism are the perfect combination for solving the centralisation problem caused by the integration of technology and entity. The vast majority of existing information systems are centred on the Internet platform. Use blockchain technology to build a decentralised business and service evaluation system. Create an open and trusted community ecology. Rewards and penalties for users and merchants will be dealt with fairly. The provider cannot falsify or tamper with transaction data. The addition of regulators will limit the release and sale of sky-high commodities and prevent arbitrary charges. It has already reduced the problems of fraud, big data killing, and technology abuse in voting comments. The new model is making tourism more comfortable, intelligent and efficient, and it is also improving the quality of tourism evaluation and diversifying tourism formats.

## 4 TM models in the context of sustainable blockchain

### 4.1 Connotation and characteristics of sustainable blockchain

Blockchain was first introduced in bitcoin in 2008, and in 2009 the first block with the number 0 was born. A few days later, a block numbered 1 appeared and was connected to the first block, forming a chain, which was the birth of the blockchain. In the creation process of bitcoin, blocks are storage units that record all communication information of each region within a certain period of time. Each block of data is linked together by a hash algorithm, and each block of data contains the hash value of the previous block of data as the information spreads. The connections between each data block become increasingly close, eventually forming a blockchain [22]. In recent years, with the rapid development of technology, blockchain has attracted increasing attention and has been widely applied in various fields. The characteristics of blockchain are as follows.

(1) The decentralisation feature. Blockchain technology employs a distributed storage and computing method that is not reliant on the involvement of third-party management agencies, nor is it subject to central regulation. The system is capable of autonomously managing, transmitting, and verifying information from disparate nodes, with each node bearing equal obligations and rights. The loss or damage of any node would not affect the continuous operation of the network, thereby ensuring a high degree of decentralisation, which is also its most essential feature.
(2) The openness feature. In the system, only the private information of each trading party is encrypted, while other data is made public. The public interface allows users to query, use, and develop the data. Consequently, the system exhibits a high degree of openness and transparency.
(3) The system exhibits characteristics of independence. The operation of blockchain systems does not rely on third parties, and it uses negotiated protocols and specifications as the basis. This is similar to Bitcoin, which uses hash algorithms. Any node is capable of exchanging and verifying data independently of the system, without being affected by human intervention, and thus exhibits strong independence.
(4) The non-tampering feature. Verification of data results in its addition to the corresponding blockchain, which is then permanently saved. Any modification of the database by a single node would have no effect, thereby ensuring the reliability and stability of this technology.
(5) The system incorporates a number of features designed to facilitate traceability. Blockchain technology employs the use of timestamps to document the sequence and comprehensive details of transactions, thereby extending the temporal scope and enhancing data traceability.

### 4.2 Traditional TM model

The traditional scenic spots are mainly focused on sightseeing tourism, which presents a number of challenges. These include a low industrial level, a limited range of products and a weak industrial foundation. The principal products of the dominant tourism industry must be enhanced to provide greater stimulation and growth. The operational framework is not yet fully developed, and the industrial chain has yet to be established [23]. A considerable number of traditional tourist attractions have received inadequate investment in infrastructure. The area in question is lacking in the requisite recreational facilities and related commercial activities. Furthermore, the absence of basic tourism supporting industries, such as hotels, department stores and entertainment venues, is a significant shortcoming. Furthermore, the absence of fundamental support facilities, such as parks and squares, renders the destination unattractive to tourists and places undue reliance on ticket revenues to sustain operations. The region has not yet established a tourism "six-element" industrial chain comprising food, housing, transportation, tourism, shopping and entertainment. The traditional management models are unable to effectively plan, direct and manage the tourism industry.

### 4.3 TM model in the context of sustainable blockchain

Blockchain technology is built on decentralised storage based on a peer-to-peer network. This core cornerstone effectively protects stored records from tampering. Blockchain is a decentralised technical solution that differs from the traditional centralized model. Any servers in the system are independent, peer-to-peer distributed nodes with the same operation rights and are interconnected in a flat topology. This ensures that participants can equally join the public chain as users or miners [24]. This is how data is stored on multiple servers through a distributed ledger. This decentralised storage architecture based on a peer-to-peer network ensures that blockchain distributes information and verifies data through the entire system, and supports fast access while ensuring data security. It significantly improves the interoperability of the system [25].

**Fig. 2:**
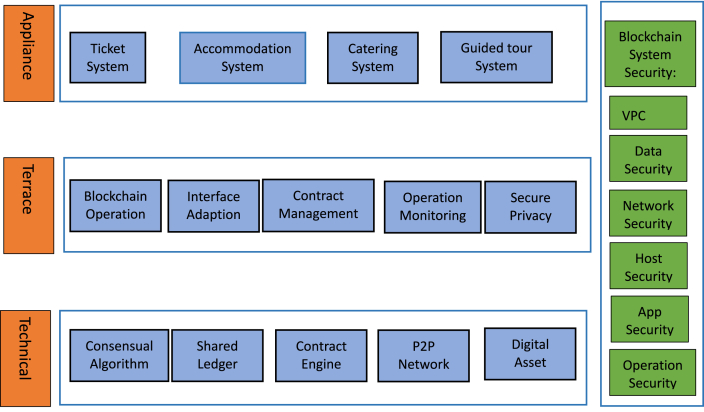
TM model.

Blockchain distributed technology [26] can be used to establish a model for tourism service management, which includes modules such as dining, accommodation, transportation, tour guide services, and others. The TM model diagram is shown in Fig. 2.

The transformation of the tourism service project module into a mathematical model is as follows:

(1) $$\begin{array}{cc} Q=\left[\begin{array}{c} \begin{array}{cc} \begin{array}{ccc} Q_{1} & {\text{qa}}_{1} & {\text{qb}}_{1}\\ Q_{2} & {\text{qa}}_{2} & {\text{qb}}_{2} \end{array} & \begin{array}{c} {\text{qflag}}_{1}\\ {\text{qflag}}_{2} \end{array} \end{array}\\ \begin{array}{cc} \begin{array}{ccc} \cdots & \cdots & \cdots \\ Q_{x} & {\text{qa}}_{x} & {\text{qb}}_{x} \end{array} & \begin{array}{c} \cdots \\ {\text{qflag}}_{x} \end{array} \end{array} \end{array}\right] & P=\left[\begin{array}{c} \begin{array}{cc} \begin{array}{ccc} P_{1} & {\text{pa}}_{1} & {\text{pb}}_{1}\\ P_{2} & {\text{pa}}_{2} & {\text{pb}}_{2} \end{array} & \begin{array}{c} {\text{pflag}}_{1}\\ {\text{pflag}}_{2} \end{array} \end{array}\\ \begin{array}{cc} \begin{array}{ccc} \cdots & \cdots & \cdots \\ P_{x} & {\text{pa}}_{x} & {\text{pb}}_{x} \end{array} & \begin{array}{c} \cdots \\ {\text{pflag}}_{x} \end{array} \end{array} \end{array}\right]\\ \begin{array}{c} S=\left[\begin{array}{c} \begin{array}{cc} \begin{array}{ccc} S_{1} & {\text{sa}}_{1} & {\text{sb}}_{1}\\ S_{2} & {\text{sa}}_{2} & {\text{sb}}_{2} \end{array} & \begin{array}{c} {\text{sflag}}_{1}\\ {\text{sflag}}_{2} \end{array} \end{array}\\ \begin{array}{cc} \begin{array}{ccc} \cdots & \cdots & \cdots \\ S_{x} & {\text{sa}}_{x} & {\text{sb}}_{x} \end{array} & \begin{array}{c} \cdots \\ {\text{sflag}}_{x} \end{array} \end{array} \end{array}\right]\\ G=\left[\begin{array}{c} \begin{array}{cc} \begin{array}{ccc} G_{1} & {\text{ga}}_{1} & {\text{gb}}_{1}\\ G_{2} & {\text{ga}}_{2} & {\text{gb}}_{2} \end{array} & \begin{array}{c} {\text{gflag}}_{1}\\ {\text{gflag}}_{2} \end{array} \end{array}\\ \begin{array}{cc} \begin{array}{ccc} \cdots & \cdots & \cdots \\ G_{x} & {\text{ga}}_{x} & {\text{gb}}_{x} \end{array} & \begin{array}{c} \cdots \\ {\text{gflag}}_{x} \end{array} \end{array} \end{array}\right] \end{array} & K=\left[\begin{array}{c} \begin{array}{cc} \begin{array}{ccc} K_{1} & {\text{ka}}_{1} & {\text{kb}}_{1}\\ K_{2} & {\text{ka}}_{2} & {\text{kb}}_{2} \end{array} & \begin{array}{c} {\text{kflag}}_{1}\\ {\text{kflag}}_{2} \end{array} \end{array}\\ \begin{array}{cc} \begin{array}{ccc} \cdots & \cdots & \cdots \\ K_{x} & {\text{ka}}_{x} & {\text{kb}}_{x} \end{array} & \begin{array}{c} \cdots \\ {\text{kflag}}_{x} \end{array} \end{array} \end{array}\right] \end{array}$$

According to the model calculation formula, the total price of the service item can be calculated as follows:

(2) Y=Q+P+S+K+G

In the formula: Y represents the total price of tourism service items; Q is the dining price standard; P is the accommodation price standard; S is the transportation standard;

K is the price standard for tour guide services; G is the price standard for other tourism service items. Among them:

$$Q=\sum Q_{r}\left(Q_{r}={\text{qa}}_{r}\times {\text{qb}}_{r}\times {\text{qflag}}_{r},r=1,\ldots ,x\right)$$

$$P=\sum P_{r}\left(P_{r}={\text{pa}}_{r}\times {\text{pb}}_{r}\times {\text{pflag}}_{r},r=1,\ldots ,x\right)$$

(3) $$S=\sum S_{r}\left(S_{r}={\text{sa}}_{r}\times {\text{sb}}_{r}\times {\text{sflag}}_{r},r=1,\ldots ,x\right)$$

$$K=\sum K_{r}\left(k_{r}={\text{ka}}_{r}\times {\text{kb}}_{r}\times {\text{kflag}}_{r},r=1,\ldots ,x\right)$$

$$G=\sum G_{r}\left(G_{r}={\text{ga}}_{r}\times {\text{gb}}_{r}\times {\text{gflag}}_{r},r=1,\ldots ,x\right)$$

Finally, an order can be generated online, and tourists can print out the order after paying to start traveling.

### 4.4 Research and application of tourism management in the Internet of Things industry

With the continuous development of IoT technology, IoT has gradually penetrated into various industries, including the tourism industry. The research and application of Internet of Things technology in tourism management can improve the management efficiency and service quality of the tourism industry, and provide tourists with a more convenient, safe and comfortable travel experience.

(1) Unified management of staff.

In terms of human resources, the recruitment, in-service and out-of-service information of each unit is updated in time to facilitate personnel scheduling. In terms of business responsibility, IoT technology is used to carry out business responsibility management, such as recording the presence of field personnel through RFID, tracking the arrival of employees, scheduling and response [27], and ultimately, the information output from IoT is used to provide personnel performance.

(2) Integrated management of the environment and facilities

After the full deployment of IOT infrastructure equipment in the scenic area, it will form an information network centred on the data centre, and through the collection of a large number of IOT equipments, GPS positioning and remote sensing, it will obtain the spatial data of the information, and process and transform the collected data from the perspective of informatization, which can play a prominent role in the resource management of scenic areas and environmental safety management and detection in particular [28]. At the same time, the background accurately records the latitude and longitude of the equipment, forming a three-dimensional 3D mapping map, networking all the IoT terminals, obtaining the spatial coordinates and carrying out dynamic management [29]. In terms of enhancing the management efficiency of scenic spots, it realises all-round real-time control and management of scenic spot monitoring, control, maintenance, security, emergency and inspection situations. Enhance real-time monitoring, timely response, rapid treatment of regulatory capacity. In the scene of real-time early warning, to deal with dangerous crowd warning, dangerous area alarm, tourists call for help in case of emergency, natural disaster warning, fire alarm processing, passenger flow over the limit of the alarm and congestion warning.

(3) Unified management of equipment operation and maintenance.

For equipment problems due to insufficient operation and maintenance capabilities, operation and maintenance management is a basic management problem, in the hope that the management unit to strengthen the sense of responsibility, improve the operation and maintenance capabilities at the same time, can still be used to improve the operation and maintenance capabilities with the help of the means of digital intelligence [30]. Construction of the "equipment operation view " for the scenic area of all the IoT equipment networking, operation monitoring, if there is equipment failure and off-network conditions, timely warning and release work orders to the responsible operation and maintenance personnel to check and repair.

## 5 Application of TM models in the context of sustainable blockchain

This paper selects 20 tourist attractions based on the TM model to verify the validity of the TM model in the context of sustainable blockchain research. (In order to facilitate the compilation of subsequent experimental analysis, 20 tourist attractions were marked as 1–20.) Table 2 shows the information on tourist attractions. We captured data on scenic spots released by the China National Tourism Administration and interviewed staff at various levels at these spots. We evaluated the TM model from four aspects: tourist satisfaction, tourism infrastructure perfection, tourism consumption level and tourism service richness. To ensure the objectivity of the experimental results, the evaluation results were compared with those of the traditional TM system.

**Table 2:** List of tourist attractions.

	Scenic spot name	Subordinate region	Grade of scenic spot
1	Xinzhou city Wutai Mountain scenic area	Shanxi	5A
2	Cloud Grottoes in Datong City	Shanxi	5A
3	Jiexiu Mianshan scenic spot in Jinzhong	Shanxi	5A
4	Longmen Grottoes scenic spot in Luoyang	Henan	5A
5	Dengfeng Songshan Shaolin scenic spot	Henan	5A
6	Central Plains Big Buddha scenic area	Henan	5A
7	Baiyun Mountain scenic spot in Luoyang	Henan	5A
8	Yin Ruins scenic spot of Anyang	Henan	5A
9	Kaifeng Qingming River garden	Henan	5A
10	Wuhan East Lake scenic spot	Hubei	5A
11	Changyang Qingjiang Gallery scenic spot	Hubei	5A
12	Three Gorges Dam tourist area	Hubei	5A
13	Yellow Crane Tower Park	Hubei	5A
14	Tianmen Mountain tourist area	Hunan	5A
15	Nanyue Hengshan tourist area	Hunan	5A
16	Orange Island tourist area	Hunan	5A
17	Changsha Huaming Building scenic spot	Jiangxi	5A
18	Lushan Scenic spot	Jiangxi	5A
19	Jiuhuashan Scenic Spot	Anhui	5A
20	Xidi Hong Village	Anhui	5A

Note: Tourist attractions in China are classified into different levels according to the laws related to the quality of tourist attractions, and there are five levels in total, namely 5A, 4A, 3A, 2A and 1A. 5A level attractions are the best.

### 5.1 Tourist satisfaction

Tourism satisfaction [31] refers to tourists’ evaluation of itinerary, attractions, accommodation, catering, shopping, time, tour guides, vehicles, and other aspects. The satisfaction of tourists with this trip to a certain extent determines the way they choose to travel next time.

**Fig. 3:**
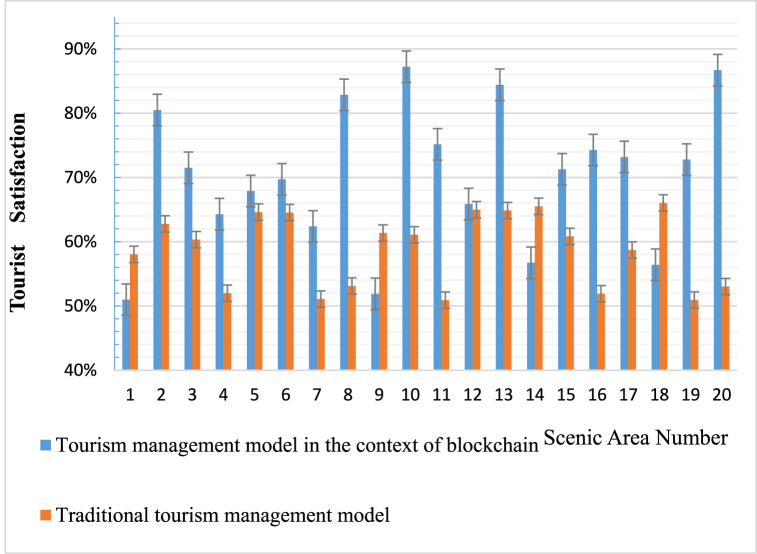
Comparison of tourist satisfaction results between TM models and traditional TM systems in the context of sustainable blockchain.

The visitor satisfaction results in this paper are based on visitors' online evaluations on the scenic area's website, and 100 valid satisfaction evaluations were randomly selected. This article compares the satisfaction evaluation results of TM models in the context of sustainable blockchain with those of traditional TM systems. The comparison diagram is shown in Fig. 3.

According to Fig. 3, the satisfaction rates of scenic spots numbered 1, 5, 10, 15, and 20 under the sustainable blockchain TM model are 51 %, 68 %, 87 %, 71 %, and 87 %, respectively. Under the traditional TM system, the satisfaction rates of scenic spots numbered 1, 5, 10, 15, and 20 are 58 %, 65 %, 61 %, 61 %, and 53 %, respectively. From the comparison of satisfaction results, it can be seen that in the context of sustainable blockchain, the TM model has been applied to 16 out of 20 scenic spots, and the satisfaction of tourists in 16 scenic spots is higher than that obtained from traditional TM systems. Its explanation is that the TM model in the context of sustainable blockchain can provide tourists with better experiences in terms of itinerary, attractions, accommodation, catering, shopping, time, tour guides, vehicles, etc., which is conducive to increasing tourists’ stickiness.

### 5.2 Perfection of tourism infrastructure

Tourism infrastructure [32] refers to various tangible facilities built to meet the tourism needs of tourists. Tourism infrastructure is an indispensable material foundation for tourism development. It mainly refers to all ground and underground structure, such as water supply, sewage treatment, air supply, power, drainage, roads, communication networks, and many commercial facilities. In order to provide convenience for tourists and guarantee tourism services, various infrastructure plays a crucial role in the construction of tourist areas. When people enter the scenic area, their first impression is that it is a large public place, and without good infrastructure, many tourists would not be able to afford it. For example, there are few and difficult to find public toilets, and there are few garbage bins on the road; The lack of road signs greatly affects the feelings of tourists.

**Fig. 4:**
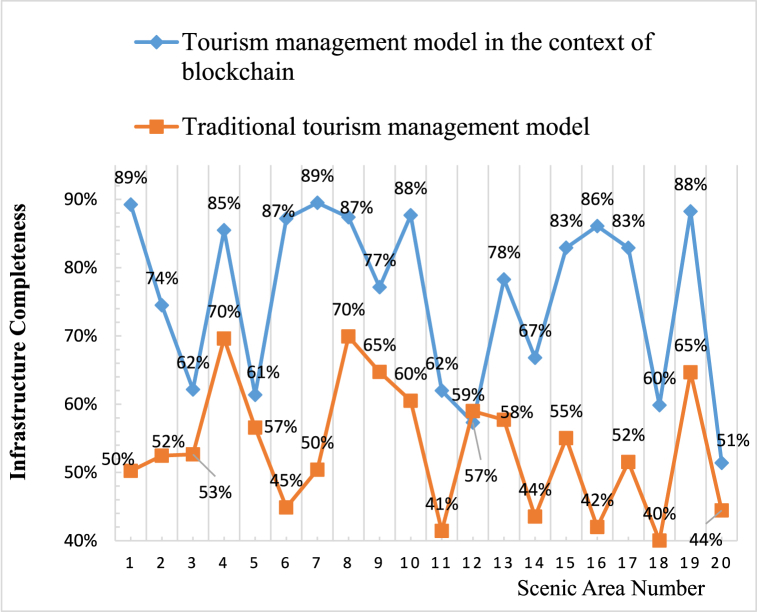
Comparison of infrastructure improvement results between TM model and traditional TM system in the context of sustainable blockchain.

The results of the completeness of tourism infrastructure in this article are obtained from maps and field investigations of various scenic spots, mainly examining infrastructure that directly affects tourists’ feelings, such as public toilets, trash cans, road signs, and commercial equipment. This article compares the infrastructure improvement results of TM models in the context of sustainable blockchain with those of traditional TM systems, as shown in Fig. 4.

From Fig. 4, it can be seen that the infrastructure completeness of the TM models numbered 1, 5, 10, 15, and 20 in the context of sustainable blockchain is 89 %, 61 %, 88 %, 83 %, and 51 %, respectively. Under the traditional TM system, the completeness of infrastructure numbered 1, 5, 10, 15, and 20 is 50 %, 57 %, 60 %, 55 %, and 44 %, respectively. From the comparison results of infrastructure completeness, it can be seen that the infrastructure construction of scenic spots under the sustainable blockchain TM model is mostly more complete than that under traditional TM. This indicates that the TM model in the context of sustainable blockchain can better plan scenic spots, provide convenience for tourists, and greatly improve their feelings.

### 5.3 Tourism consumption level

The level of tourism consumption refers to the satisfaction and level of the quantity and demand of tourism products and services purchased by tourists during the tourism process. Narrowly defined “tourism consumption” refers to the per capita consumption of tourists in tourism products; In a broad sense, the level of tourism consumption not only refers to the per capita expenditure on tourism, but also refers to the quality and level of people's consumption of tourism products. This also reflects the degree and level of satisfaction of tourists with their tourism needs. From the perspective of consumption, tourism consumption refers to a psychological feeling and evaluation of tourists towards tourism products. Due to the strong serviceability of tourism products, tourism consumption is a spiritual consumption of tourists towards the services they provide. Meanwhile, as time goes by, the needs of tourists also change accordingly. So, on the one hand, tourism enterprises must comply with certain standards and requirements, and provide tourists with corresponding tourism products and services. On the other hand, it is also necessary to continuously adjust and improve the quality of tourism services based on tourists' consumption feedback.

**Fig. 5:**
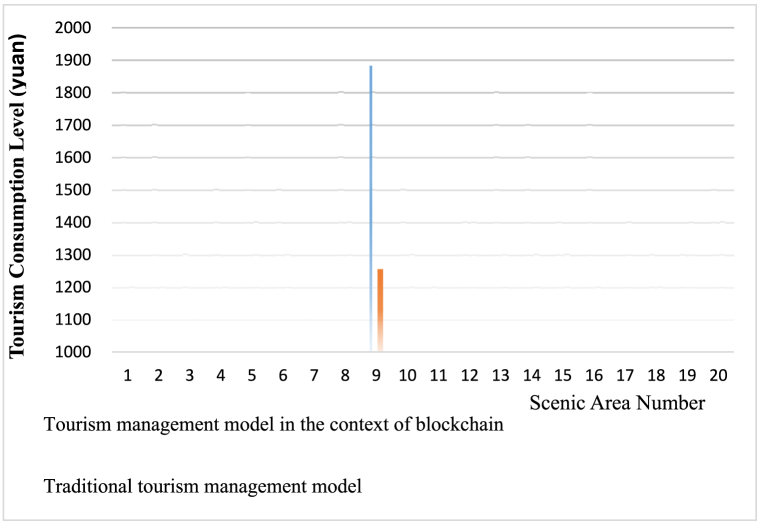
Comparison of tourism consumption levels between TM models and traditional TM systems in the context of sustainable blockchain.

The tourism consumption level of this article is calculated based on the turnover of each scenic area during a certain period of time and the total number of tourists. The turnover here includes the ticket turnover and the turnover of all consumption facilities and services within the scenic area. This article compares the tourism consumption level of TM models in the context of sustainable blockchain with that of traditional TM systems. The comparison diagram is shown in Fig. 5.

By comparing Fig. 5, it can be seen that the tourism consumption levels for TM models numbered 1, 5, 10, 15, and 20 in the context of sustainable blockchain are 1814 yuan, 1799 yuan, 1565 yuan, 1483 yuan, and 1539 yuan, respectively. Under the traditional TM system, the tourism consumption levels numbered 1, 5, 10, 15, and 20 are 1292 yuan, 1418 yuan, 1353 yuan, 1337 yuan, and 1430 yuan, respectively. From the comparison of tourism consumption levels, it can be seen that the tourism consumption level of scenic spots under the sustainable blockchain TM model is mostly higher than that under traditional TM. Its explanation is that the TM model in the context of sustainable blockchain can better serve tourists, meet their demand for tourism, and stimulate their consumption.

### 5.4 Abundance of tourism service content

Tourism service refers to the use of various facilities, equipment, methods, and means by tourism service providers to create a harmonious atmosphere and create a spiritual psychological effect to stimulate tourists’ emotions through various expressions of “hospitality”. It resonates with the hearts of tourists, allowing them to experience a sense of satisfaction and happiness while enjoying this service, and to be willing to communicate with the service provider for consumption [33,34]. The scope of tourism services is very extensive, and the specific content includes the following aspects.

(1) It provides tickets for tourists and guides them on how to use them.
(2) It enables tourists to quickly and efficiently find the scenic spots they want to visit.
(3) It tells tourists where it is safe and where it is dangerous, ensuring the safety of their lives.
(4) It introduces the local culture to tourists, allowing them to fully appreciate the unique charm of this scenic area.
(5) It provides catering, leisure, entertainment and other services for tourists.
(6) It meets the needs of tourists for taking photos as a souvenir.
(7) When tourists encounter abnormal conditions, staff should provide timely assistance.

**Fig. 6:**
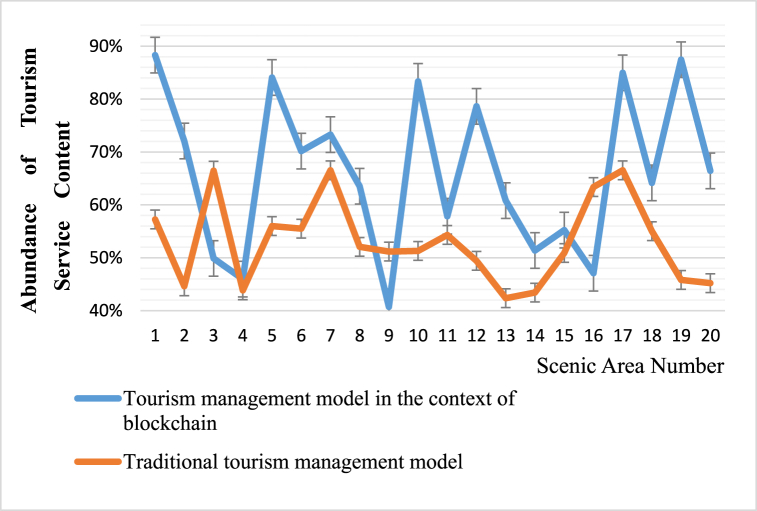
Comparison of the richness of tourism service content between TM models and traditional TM systems in the context of sustainable blockchain.

The richness of tourism service content in this article is evaluated from several aspects of tourism services in the scenic area instruction manuals of each scenic area. This article compares the richness of tourism service content oriented towards sustainable blockchain TM models with that of traditional TM systems. The comparison diagram is shown in Fig. 6.

According to Fig. 6, the richness of tourism service content numbered 1, 5, 10, 15, and 20 in the context of sustainable blockchain management is 88 %, 84 %, 83 %, 55 %, and 66 %, respectively. Under the traditional TM system, the richness of tourism services numbered 1, 5, 10, 15, and 20 is 57 %, 56 %, 51 %, 51 %, and 45 %, respectively. From the comparison results of the richness of tourism service content in this article, it can be seen that the richness of tourism service content in scenic areas under the sustainable blockchain TM model is mostly higher than that under traditional TM. Its explanation focuses on the importance of tourism services in the context of sustainable blockchain management, creating a harmonious atmosphere for tourists and providing them with a better experience in tourism.

## 6 Conclusions

This article presents a comprehensive examination of the potential applications of sustainable blockchain technology in the field of tourism management. A tourism management system is designed based on blockchain and the Internet of Things. Furthermore, a comparison is presented between the evaluation results of TM models applied in scenic areas and those of traditional TM models in the context of sustainable blockchain. It is indubitable that blockchain technology will facilitate the creation of more collaborative business models, the simplification of the flow of information, and the collaboration of travel companies and stakeholders in the management of customer loyalty. Blockchain technology will facilitate the sharing of tourist information between tourism enterprises, enhance tourist satisfaction, and enable the establishment of mutually beneficial business collaboration models to promote cooperation between tourism destination enterprises. The construction of an optimal tourism commercial application system represents a significant challenge for the future of tourism blockchain technology. The objective is to ascertain whether the research advantages of blockchain technology can be transformed into practical applications, with a view to optimising the structure of the tourism industry.

This study's limitations and future direction are twofold. Firstly, the application of blockchain technology in tourism is still in its infancy, and the challenge of rapidly developing a comprehensive tourism business application system is a significant one for the future development of tourism blockchain technology. Secondly, while the research and development of tourism blockchain basic technology is paramount, it is also essential that tourism enterprises are actively guided to reshape their internal organisational structure and culture. The establishment of a culture of knowledge sharing and collaboration within the enterprise is of great significance for the eventual application of blockchain technology in the enterprise.

## Funding

This work was supported by the Jiangsu Province Education Science planning project (B20220292); Hangzhou Philosophy and Social Science Planning Project (2024JD055).

## CRediT authorship contribution statement

**Zhenhua He:** Writing – original draft, Visualization, Validation, Resources, Methodology, Conceptualization. **Wenwen Yu:** Validation, Investigation, Formal analysis. **Lifeng Chen:** Writing – review & editing, Supervision, Software, Project administration, Investigation, Data curation.

## Declaration of competing interest

The authors declare that they have no known competing financial interests or personal relationships that could have appeared to influence the work reported in this paper.
